# The Force Synergy of Human Digits in Static and Dynamic Cylindrical Grasps

**DOI:** 10.1371/journal.pone.0060509

**Published:** 2013-03-27

**Authors:** Li-Chieh Kuo, Shih-Wei Chen, Chien-Ju Lin, Wei-Jr Lin, Sheng-Che Lin, Fong-Chin Su

**Affiliations:** 1 Department of Occupational Therapy, National Cheng Kung University, Tainan, Taiwan; 2 Department of Biomedical Engineering, National Cheng Kung University, Tainan, Taiwan; 3 Medical Device Innovation Center, National Cheng Kung University, Tainan, Taiwan; 4 Section of Plastic Surgery, Department of Surgery, National Cheng Kung University, Tainan, Taiwan; The University of Queensland, Australia

## Abstract

This study explores the force synergy of human digits in both static and dynamic cylindrical grasping conditions. The patterns of digit force distribution, error compensation, and the relationships among digit forces are examined to quantify the synergetic patterns and coordination of multi-finger movements. This study recruited 24 healthy participants to perform cylindrical grasps using a glass simulator under normal grasping and one-finger restricted conditions. Parameters such as the grasping force, patterns of digit force distribution, and the force coefficient of variation are determined. Correlation coefficients and principal component analysis (PCA) are used to estimate the synergy strength under the dynamic grasping condition. Specific distribution patterns of digit forces are identified for various conditions. The compensation of adjacent fingers for the force in the normal direction of an absent finger agrees with the principle of error compensation. For digit forces in anti-gravity directions, the distribution patterns vary significantly by participant. The forces exerted by the thumb are closely related to those exerted by other fingers under all conditions. The index-middle and middle-ring finger pairs demonstrate a significant relationship. The PCA results show that the normal forces of digits are highly coordinated. This study reveals that normal force synergy exists under both static and dynamic cylindrical grasping conditions.

## Introduction

A well-controlled integration of the musculoskeletal system, neural system, and cutaneous sensations is essential for performing skilled finger movements [1]. In daily life, manipulating objects usually involves multi-finger coordination [2]. However, few people in daily life consider how to handle a given object appropriately with the correct digit interactions in a given situation. According to the concept of minimal disturbance, each part of the hand moves efficiently while manipulating an object. Bernstein hypothesized that synergies may simplify the coordination of muscles [3]. Digit synergy is defined as interdependent movements among digits, and it provides high stability in the coordination of the spaces of elemental variables during performance [4]. The muscle synergies of multi-digit movements have been studied from both kinematic and kinetic perspectives [5]. Synergetic control can be derived from either anatomical or neural mechanisms. From an anatomical perspective, a single muscle can be inserted into multiple points of a hand or across multiple joints. For example, the flexor digitorum superficialis arises from the medial epicondyle of the humerus, crosses the carpal tunnel, and divides into four tendon ends. The tendon ends are inserted into the lateral border in the center of the middle phalanges of the second to fifth fingers. Thus, during muscle contraction, four fingers flex simultaneously. Additionally, the projection areas of the fingers in the primary motor cortex exhibit substantial overlaps [6]. The overlapping digit areas in the motor cortex suggest a neural contribution to the synergetic movement of digits.

A cylindrical grasp is an advanced multi-finger-controlled movement. The primary agonists of this action are finger flexors, such as the flexor digitorium superficialis, flexor digitorium profundus, and some intrinsic muscles [7], [8], [9], [10]. Virtual finger (VF) theory describes the configuration of fingers when grasping a cylindrical object [11]. When holding an object using five digits, four fingers become a single VF that produces forces opposite to those of the thumb to hold the object. The thumb force counterbalances the force produced by the VF. A previous study found that forces from the index and middle fingers act against the force produced by the thumb during the three-fingered tripod grasp, which supports VF theory [12]. VF theory also holds under conditions of the grasp torque being altered or the shape of the holding object being changed, and thus can be regarded as related to the concept of synergy. Synergies in hand kinetics have also been found for multi-digit movements, such as sharing force and error compensation [13], with the former being the percentage of digit force in the total manipulation force [14], [15]. A recent study also used VF theory by analyzing how the forces exerted by the fingers are modified by the additional action of the thumb during the crimp grip, and concluded that the central nervous system organized the association of all the digits [16]. In a specific hand movement, it is assumed that there is a constant grasp force distribution pattern among individuals. Error compensation can be considered a basic characteristic of motor synergies [17], and is observed when one or more fingers are unable to contribute to performing a task. When a finger is restricted from participating in a parallel pressing task [13], the force produced by the remaining digits increases substantially, because the central controller tries to maintain the movement with minimal disturbance.

A previous study used different quantitative methods to explore mechanical and neural functions during various manipulation tasks [18], although the coordination parameters were not sufficiently precise to explain the process of hand coordination. Correlation coefficients and principal component analysis (PCA) are used to examine the synergy strength of performance variables, such as joint angles and finger forces. However, few studies have focused on functional multi-finger movements, such as panel-like finger movements or handle-like grasping movements [19], [20]. This study measures the force distribution patterns among digits based on the sharing force concept during cylindrical grasping. Generally, a grasping force is distributed in a specific sharing pattern, which can be considered muscle synergy or force synergy, that can then be used to differentiate between healthy and impaired people [21]. Error compensation, where an adjacent finger undertakes the work of a missing one, has been demonstrated in the grasping and parallel pressing tasks. Unlike previous movement tests, this study attempts to use a more functional perspective to comprehend the roles of digits under a cylindrical grasping task. This study thus aims to investigate the kinetic performances of the cylindrical grasp using a custom glass simulator and the concept of error compensation. It is hypothesized that by using this approach the force distribution patterns, grasp stability and coordination among fingers can be explained for both normal and finger-restricted grasps.

## Materials and Methods

### Participants

Twenty-four healthy participants (12 men and 12 women) with no history of hand injuries or sensory deficits were recruited in this study. All participants were informed about the purpose of the study and signed consent forms. The study was approved by the Institutional Review Board (no. ER-99-112) of National Cheng Kung University Hospital. Their mean age was 26, and all participants were right-handed.

### Apparatus

**Figure 1:**
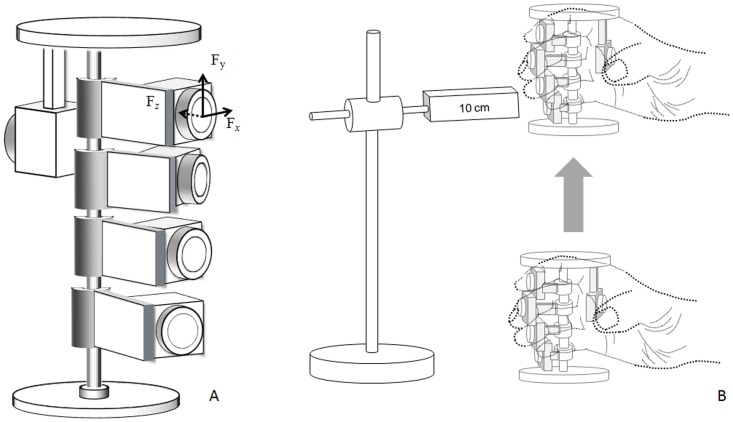
Experimental apparatus and testing procedure. (A) Glass simulator equipped with five force transducers. Each force transducer had its own force coordination system. *Fx*: tangential force vector in the clockwise direction; *Fy*: force vector along the central axis of the glass simulator in the anti-gravity direction; *Fz*: axial force toward the center axis of the glass simulator. (B) Cylindrical grasping with five fingers.

A custom-designed glass simulator (weight  =  430 g, diameter  =  6.8 cm, height  =  9 cm; Fig. 1A) mounted with five six-component force-moment transducers (one Nano25 and four Nano17; ATI Industrial Automation, Apex, NC, U.S.A.) was used to record the applied loads of digits at a sampling rate of 100 Hz. The positions of the force-moment transducers were adjusted to fit each participant’s natural posture. The analog signals were transmitted to a 12-bit analog-to-digital converter (A/D, GW Instruments, Inc., Somerville, MA, U.S.A.) and stored on a laptop using a PCMCIA card.

### Procedures

Participants were instructed to sit upright facing a desk, with their testing arm supported by the desk. The glass simulator was positioned in front of the participant at a distance of half the length of the participant’s upper extremity. During the test, participants were instructed to cylindrically grasp the simulator with only the finger pads contacting the force-moment transducers (Fig. 1B), vertically lift it at a comfortable speed to a height of 10 cm above the desk surface with the aid of a vertical bar with a height indicator, and then maintain this posture for 10 s. Five experimental configurations were randomly assigned to the participants: normal, T-MRL, T-IRL, T-IML, and T-IMR conditions (T: thumb; I: index finger; M: middle finger; R: ring finger; L: little finger). In the normal condition, the participants were asked to hold the glass simulator with five digits; in the other four finger-restricted conditions, participants held the glass in the same way as in the grasp of normal condition, but with one finger restricted in order to simulate subjects with a missing finger. The restricted finger was passively flexed and fastened with elastic adhesive tapes to maintain the distal and proximal interphalangeal joints in full flexion, and the metacarpophalangeal joint in an unrestrained condition. For example, in the T-MRL condition, the index finger was restricted and the participant had to grasp the glass simulator with the thumb, middle finger, ring finger, and little finger. For each condition, the task was repeated five times, with a 3-min rest between each trial.

### Data analysis

The data were processed using custom-made Matlab programs (MathWorks, Natick, MA, USA) to filter noise and calculate the parameters. The entire task was divided into several phases, with the force increasing phase (FIP) and the holding stable phase (HSP) being the two main ones considered in this work. The FIP is the period from 10% to 100% of the peak grasping force, and the HSP is a 3-s period following the FIP. During the HSP, the participants maintained the glass simulator in a stable position, and so the grasping force at this time exhibited low variability.HSP can thus be considered a static situation, whereas FIP is a dynamic one.

The applied forces were divided into three orthogonal components, namely *Fx*, *Fy*, and *Fz*. *Fy* represents the force vector parallel to the long axis of the glass simulator in the anti-gravity direction. *Fz* represents the force vector toward the center of the glass simulator. *Fx* is normal to both *Fy* and *Fz*. The resultant force of each digit is the synthesis of *Fx*, *Fy*, and *Fz*. The summation of each digit’s force (*F_T_, F_I_, F_M_, F_R_,* and *F_L_*) is represented as the total grasping force (*F_G_*). The mean value of the total grasping force and digit force in the HSP were adopted to compute the force contribution patterns in the HSP. The force contribution pattern was calculated as each digit force divided by the total grasping force. The coefficient of variation (CV) of the digit force and the total grasp force in the HSP was analyzed to determine the disturbance or variability of the contribution of the digit force to the total grasp force. CV is a commonly used method of representing the stability or smoothness of a movement or performance. Force-stabilizing synergies have been observed in multi-finger force production [22], with the central controller appearing to maintain the total force under stable conditions.

The *Fy* and *Fz* components of the digit forces in the FIP were analyzed using correlation coefficients and PCA to examine the strength of coordination among the temporal parameters of digit force. The linear correlation coefficients were calculated for the thumb-index (TI), thumb-middle (TM), thumb-ring (TR), thumb-little (TL), index-middle (IM), index-ring (IR), index-little (IL), middle-ring (MR), middle-little (ML), and ring-little (RL) pairs. Higher correlation coefficients indicate a stronger correlation between two digit forces. In addition, PCA, an advanced statistical and mathematical method for simplifying complex biomechanical systems, was applied to detect the reasons for these force variables in the time domain. This method divided the correlated variables into smaller groups of new uncorrelated ones, called principal components (PC). The first PC explains the majority of variance of the original data. The relative contribution of each element of a PC can be considered as the weight that corresponds to a particular variable [23]. After reducing the dimensionality of the dataset, the new, meaningful underlying variables can provide useful information about the level of coordination.

### Statistics

Statistical analyses were executed using SPSS 17. The parameters in each finger-restricted condition were compared with those of a normal grasp using paired *t* tests. The differences among the roles of the digit forces were examined using one-way analysis of variance (ANOVA). The coefficient correlation test was then used for the forces between two digits in different grasp conditions. The PCA was also processed in SPSS under the following criteria [24]: (1) one set of PCs, from PC1 to PC5, had its percentage of variance accounted for (PVAF). PC1 was used to account for most of the variability of the original data, and a higher value of PC1 was considered to indicate superior synergetic performance among digit forces; and (2) five sets of PC weights were used, one for each PC. The contributions of each digit force were thus examined, and the results for the first PC and the weights of the five force variables of PC1 are discussed in the next section. The level of statistical significance was set at *p* < 0.05.

## Results

### Grasping force and force variance

**Figure 2:**
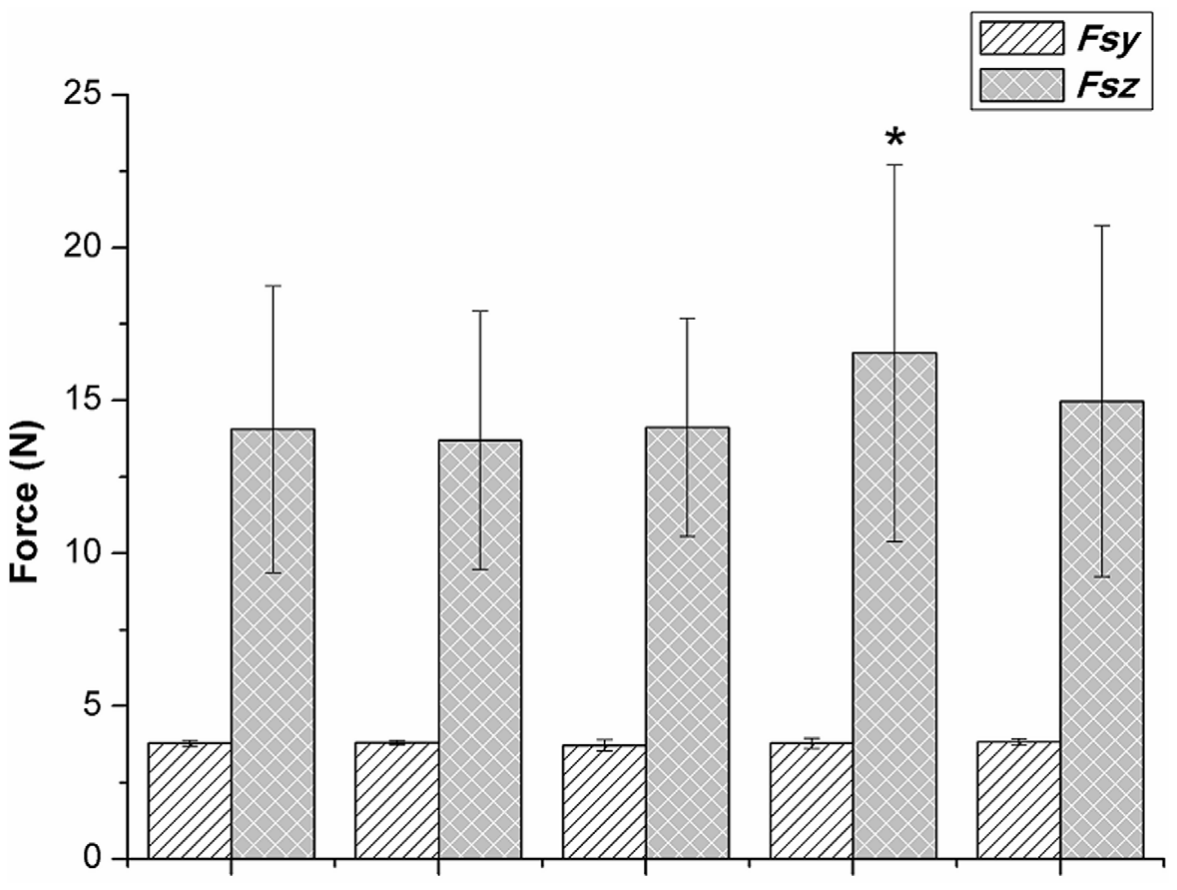
The average in the summation of the *Fy* and *Fz* components of all digit forces (*Fsy* and *Fsz*) for the normal various finger-restricted grasps for all participants. (* indicates the *Fsz* and *F_G_* in the T-IML trial were statistically greater than those in the normal grasp condition via paired *t* test).

The summation of *Fy* and *Fz* components (*Fsy* and *Fsz*) of all digit forces for the various conditions are shown in Figure 2. The *Fz* components of the contacting digits were the primary contributor of grasping force (> 80%), and the *Fy* component in all the tasks was approximately equal to the apparatus weight. Among all finger-restricted tasks, only *Fsz* and *F_G_* in the T-IML trial were significantly greater than those in the normal grasping trial, based on the results of paired *t* tests. Regarding variations in force, the normal trial was expected to produce the smallest CV value. However, the T-MRL, T-IRL, T-IML and T-IMR trials had smaller CV values for the digits than that of the normal grasping trial in the HSP, except for the values of the ring and especially the little fingers. The CV of each digit force was also analyzed using one-way ANOVA. The results show that the little finger had the statistically highest CV values in the normal and T-MRL, T-IRL, and T-IML finger-restricted grasping conditions (Table 1).

**Table 1:** CV results of each digit and different grasp conditions from this study and a previous one [19].

	CV_T	CV_I	CV_M	CV_R	CV_L	*p-*value	Post-hoc
Normal	3.6(1.3)	3.7(1.6)	4.2(2.0)	4.8(2.1)	7.1(3.7)	0.00***	CV_T < CV_L*CV_I < CV_L*CV_M < CV_ L*CV_R < CV_ L*
T-MRL	3.0(1.2)	--	2.9(1.4)	3.7(2.1)	6.1(2.1)	0.00***	CV_T < CV_L*CV_M < CV_L*CV_R < CV_L*
T-IRL	3.2(1.2)	2.9(1.1)	--	4.9(2.2)	9.0(7.2)	0.00***	CV_T < CV_L*CV_I < CV_L*CV_R < CV_L*
T-IML	3.1(1.1)	3.2(1.4)	3.8(1.6)	--	8.3(3.8)	0.00***	CV_T < CV_L*CV_I < CV_L*CV_M < CV_L*
T-IMR	2.8(1.0)	3.6(1.4)	3.7(1.9)	4.4(3.2)	--	0.08	--
Li’s study [19]	7.0(3.0)	10.0(3.7)	9.0(5.2)	7.7(3.9)	10.8(3.3)	--	--

Values represented as Mean (SD).

Statistical tests: one-way ANOVA (*** indicates with statistical significance among groups via One-way ANOVA); post-hoc: Bonferroni’s *t*-test (* indicates with statistical significance between groups via Bonferroni’s *t*-test).

CV: coefficient of variation.

CV_T: coefficient of variation of the thumb.

CV_I: coefficient of variation of the index finger.

CV_M: coefficient of variation of the middle finger.

CV_R: coefficient of variation of the ring finger.

CV_L: coefficient of variation of the little finger.

### Digit force distribution pattern

**Figure 3:**
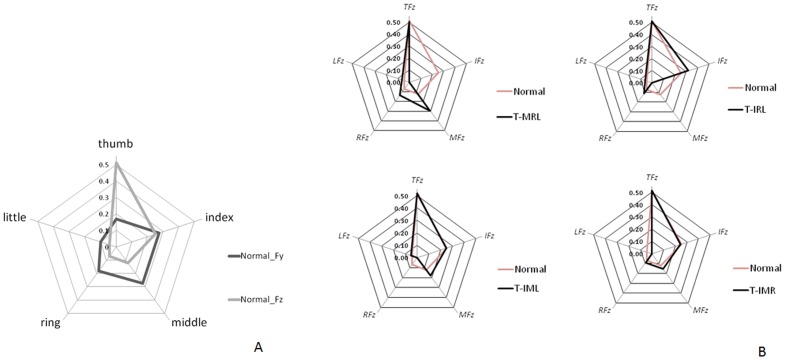
Force distribution pattern of the digits. (A) Distribution patterns for *Fy* and *Fz* of a normal grasp posture in the holding stable phase (HSP) from the average data of all participants. (B) Radar plot for comparing the distribution patterns of the force in the *Fz* direction in finger-restricted trials and normal grasp condition. (*TFz*: *Fz* of thumb. *IFz*: *Fz* of index finger. *MFz*: *Fz* of middle finger. *RFz*: *Fz* of ring finger. *LFz*: *Fz* of little finger).

The *Fy* and *Fz* components during normal grasping exhibited diverse distribution patterns, as shown in the radar plots (Fig. 3A). The thumb accounted for 50% of the grasping force in the *Fz* direction (Table 2). The *Fz* distribution is more suitable for explaining the test performances using VF theory. The force distribution patterns changed due to the finger restrictions (Fig. 3B). Relative to the *Fz* distribution, the *Fy* distribution patterns in normal postures had a greater variation (Table 2). Therefore, no specific distribution patterns were found for *Fy* because of the great variability among participants. Compared to the normal grasping condition, the contribution of the *Fz* component of the index finger increased in the T-IRL condition to compensate for the middle finger. The *Fz* component of the middle finger was increased by almost 20% in the T-MRL task to compensate for the index finger (Fig. 3B).

**Table 2:** Distribution patterns of *Fy* and *Fz* in normal grasping posture.

	Thumb	Index	Middle	Ring	Little
Fy	0.17 (0.15)	0.27 (0.13)	0.28 (0.10)	0.18 (0.08)	0.10 (0.05)
Fz	0.51 (0.01)	0.25 (0.05)	0.12 (0.04)	0.07 (0.02)	0.04 (0.02)

Values represented as Mean (SD).

### Strength of coordination

The correlation coefficients for *Fz* and *Fy* demonstrated significant differences. The *Fy* components of the digits demonstrated moderate correlation (r < 0.8) in all conditions. However, the *Fz* components of digits were highly correlated in several conditions (Table 3). For example, the thumb and index finger were always highly correlated in all conditions (r > 0.95). Similar results were also observed in the T-M, T-R, I-M, and M-R groups (r > 0.8).

**Table 3:** *Fz* correlation coefficient for two digits in different posture trials during the FIP via the linear correlation test.

	Normal	T-MRL	T-IRL	T-IML	T-IMR
Thumb vs. Index	0.97 (0.02)	--	0.98 (0.02)	0.97 (0.03)	0.97 (0.03)
Thumb vs. Middle	0.93 (0.04)	0.97 (0.02)	--	0.89 (0.12)	0.92 (0.13)
Thumb vs. Ring	0.82 (0.15)	0.90 (0.12)	<0.8	--	0.85 (0.17)
Thumb vs. Little	0.79 (0.15)	0.81 (0.16)	<0.8	<0.8	--
Index vs. Middle	0.86 (0.09)	--	--	0.8 (0.21)	0.83 (0.18)
Index vs. Ring	<0.8	--	<0.8	--	<0.8
Middle vs. Ring	<0.8	0.81 (0.18)	--	--	0.84 (0.12)
Ring vs. Little	<0.8	<0.8	<0.8	--	--

Values represented as Mean (SD).

**Figure 4:**
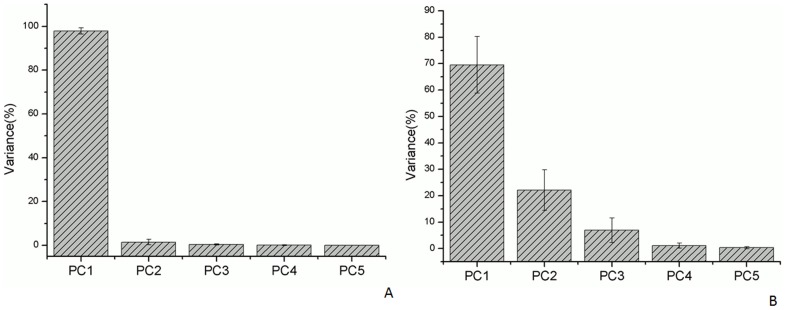
The results of PCA. (A) For *Fz* of five digits in normal grasp posture: PC1 accounted for 97% of PVAF. (B) For *Fy* of five digits in normal grasp posture: PC1 accounted for 70% of PVAF.

**Figure 5:**
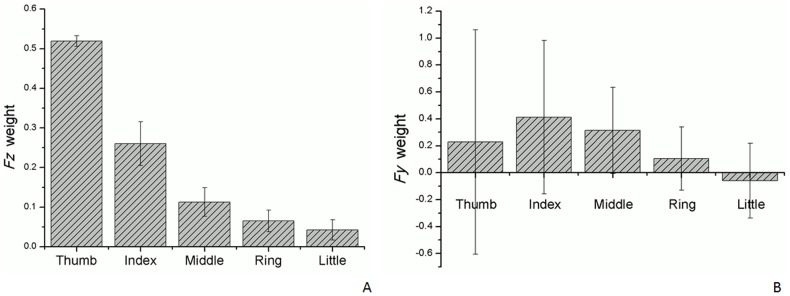
The contributions of each digit force for the first PC. (A) *Fz* components of PC1, showing a relatively consistent pattern. (B) *Fy* components of PC1, showing great variability.

The first principal component (PC1) accounted for as much of the variability in the original data as possible, and each succeeding component accounted for as much of the remaining variability as possible. *Fy* and *Fz* were analyzed separately. The PC1 of *Fy* and *Fz* accounted for almost 70% and 97% of the PVAF, respectively (Fig. 4). The thumb and index finger were the two main factors influencing the force production in the *Fz* direction, accounting for 52% and 26% of the weight, respectively (Fig. 5A). However, the PC1 composition patterns of *Fy* and *Fz* were rather varied. The value of every variable in PC1 had a greater variability in *Fy* than in *Fz* (Fig. 5B). For example, the thumb’s influence on the performance of *Fy* varied considerably, and could be positive or negative in value. The PC1 composition patterns of *Fy* and *Fz* obtained in this work were compared with those from the few other studies which have applied PCA methods to the kinematic data of hand joints (Table 4). Therefore, the PCA results for *Fz* are appropriate for assessing coordination performance.

**Table 4:** PC1 values in different studies.

Variables	Same direction§	PC1
Joint angles of thumb*	No	76%
MCP joint angles of index to little finger**	Yes	94.7∼97.3%
*Fy* of five digits (anti-gravity force) #	No	70%
*Fz* of five digits (normal force) #	Yes	98% (94.5–99.7)

* Li (2007).

** Braido (2004).

# Present study.

§ Although the variables are in the same direction, the PC1 value would a good relationship among the variables.

## Discussion

### Grasping force in cylindrical grasp

According to the results, there are no obvious changes in grasping force in the different trials. Previous studies that conducted slip tests found that people usually sustain a minimum total grasp force or maintain an adequate slip safety margin to prevent the handling apparatus slipping in the holding condition of a two- or three-digit grasp [25], [26], [27], [28]. Although few investigations have been carried out regarding the minimum grasp force required to prevent slipping with a five-digit grasp, this study assumes that the central control system attempts to maintain a stable total cylindrical grasping force and sustain the minimal performing force to hold the object, because of the force-stabilizing synergies which were described in these earlier studies. However, all performing digits during the T-IML trial increased their *Fz* magnitude to compensate for the ring finger’s absence, and the increased force reached 16% of the total grasp force in the normal trial. These results may provide surgeons with new ideas for finger reconstruction surgery. According to modern clinical suggestions, the first priority in digit reconstruction is the thumb, and the second priority is the middle or index finger, with these views based on hand functions following digit reconstruction. However, the importance of the ring finger has not been emphasized because of reliance on rough clinical measurements. Future studies should thus test more digit movements to examine the importance of each digit during specific movements.

### Force variability

The force variance, or the smoothness as well as the stability of the performance, can be used to describe finger coordination during a grasp task. The ring and little fingers are expected to have higher CV values than the thumb and index finger, because they have less individual control training. A previous study reported higher digit CV forces than those obtained in this study [19], although it analyzed the digit force CVs over the entire experiment, not only during the HSP (Table 1). However, we did not expect that the CV results of digit force in most of the finger-restricted conditions would present a smaller force variance than in the normal trial, except for the ring and especially the little fingers. Based on earlier studies, their performance in the normal trial should be equal or superior to their performance in finger-restricted trials. Therefore, this parameter’s ability to represent the smoothness (or stability) of hand performance in this study is questionable.

### Synergy difference between the normal force vector and anti-gravity force vector

#### Force distribution pattern

The VF model has been used to depict the kinetic role and inter-digit relationship of the thumb [16] and fingers in different experimental settings [12], [13], [14], [19]. The digit force distribution pattern differed between the *Fy* (anti-gravity force) and *Fz* (normal force) representations. The *Fz* distribution pattern was similar to the one proposed by Arbib [11]. For *Fz*, the thumb accounted for nearly 50% of the grasping force in all trials. During the HSP, the thumb provided a counterbalance to the remaining digits on the opposing side. The thumb’s contribution was relatively constant (50 to 52%) during all tasks, because it was counterbalancing a similar mechanical torque and force. *Fy* can be considered a friction force that is influenced by *Fz*. However, this study observed varying *Fz* and *Fy* distribution patterns among digits. Therefore, *Fz* was not the only factor influencing *Fy*, as the friction index of a digit also affects it. Here it should be noted that the digits might have different friction indices. Furthermore, the participants might have had different finger pad conditions, thus producing individual *Fy* distribution patterns.

Error compensation was found for normal force, and this is a self-regulation strategy where the adjacent fingers perform the work of a restricted one. Previous research [29] indicates that the fingers adjacent to an injured finger are most affected in terms of kinematic and kinetic performance. During this study, holding the apparatus while restricting one specific finger led the adjacent fingers to produce a greater force to hold the object. However, the adjacent fingers are not the only ones that undertake additional work. For example, a middle finger restriction caused the index, ring, and little fingers to increase their *Fz* contributions during the T-IRL trial. However, error compensation might be insufficient to explain the changes in distribution caused by finger restriction. It can thus be inferred that restricting the index or middle fingers has the greatest influence on the remaining digits, because they provide significant force contributions. Furthermore, regarding the relationships among the middle, ring, and little fingers during the T-IML, T-IMR, and T-IRL trials, the results show that the middle, ring, and little fingers comprise a special group, as each of these can increase its *Fz* contribution substantially. Therefore, the three fingers are relatively flexible in their force contributions. In other words, the results demonstrate that these three fingers could self-regulate their role to accommodate different hand situations. According to previous results [30], the ring finger is considered the most enslaved one, meaning that it is the most dependent, and can thus self-regulate its role to facilitate significant finger coordination. The middle, ring, and little fingers were found to be interdependent during finger grasping movements. From a hand rehabilitation perspective, the modulation ability of these three digits can be considered a clinical evaluation landmark, and so this finding on force synergy can be applied to develop better digit rehabilitation activities.

As mentioned previously, an *Fy* distribution pattern typically has considerable variability, and it can thus be inferred that this pattern is inconsistent in the anti-gravity direction during cylindrical grasping movements. The force distribution synergy in the *Fy* direction is difficult to explain using the error compensation concept. The index and middle fingers are the main *Fy* providers. Although *Fy* is a well-defined force vector in the anti-gravity direction, and the key force lifting the apparatus, in this study might be insufficiently sensitive to detect the force distribution synergy. Therefore, future studies should use a heavier testing apparatus to obtain the *Fy* force distribution synergy.

#### Intensity of digit force coordination

This study used correlation coefficients and PCA to evaluate the strength of coordination. Inter-digit dependence was observed among digits in the FIP. This inter-digit force tendency is considered a type of force synergy. The correlation coefficient results indicate that a similar tendency may have existed in some specific digit pairs. However, no difference in correlation coefficient was found between the finger-restricted and normal trials. Furthermore, it is difficult to detect the synergy pattern from the correlated results of a digit pair in *Fy*, due to the great variation in performance among participants. For *Fz*, the thumb demonstrated a good relationship with the index, middle, and ring fingers during all trials. The little finger is considered an isolated digit, because it is rarely well-correlated with the other fingers during either the normal or finger-restricted trials. However, the index-middle finger and middle-ring finger pairs demonstrated a fairly good relationship (*r* > 0.8). Adjacent fingers may have greater correlation and synergetic performance with one another because of anatomical and neural factors. This result agrees with both the principal and causes of synergy patterns. A previous study found a correlation between the normal force of the thumb and the sum force of the rest of the fingers in handle-holding postures [19]. The median correlation value among the participants was 0.941 in this earlier work, although the correlation values between the thumb and individual fingers were not given. Furthermore, the previous study did not analyze the performance during specific periods, such as the lifting and holding phases. Based on the results in the current study, the correlation value of two individual digit forces might provide more suitable information to describe the force synergy in a specific time period.

Only a few studies have applied PCA methods to the kinematic data of hand joints. For example, data on the thumb joint angle in the opposition movement have been analyzed using PCA [31], and the results showed that thumb joints, including the carpometacarpal, metacarpophalangeal, and interphalangeal joints, are coordinated with each other, as all joint angles in the thumb joints presented a PC1 with over 76% variance. Another study used PCA to survey the synergistic motion of finger joints from the index finger to the little finger in cylindrical movements [24]. The PC1 of all performing digits was 94.7 to 97.3%. This mathematical modeling method can characterize coordinative patterns and provide more direct and physically meaningful representations of synergistic behavior. The results of PC1 for *Fy* and *Fz* indicate variances of almost 70% and 98%, respectively. Highly synergetic digit forces can be explained using PCA, and this study concludes that *Fz* is the force vector with the highest coordination among the five digits in normal cylindrical grasp movements. Moreover, the mean PVAF of *Fz* ranged from 94.5 to 99.7%, and these results are similar to the kinematic data for the finger joint angles. Although the first two PCs of *Fy* accounted for over 90% of the PVAF, completely representing the original dataset with five force variables, *Fy* is considered to have fewer coordination patterns than *Fz*. A comparison of the PCA results with those of previous studies indicates that the variables in a given direction should have a high PC1 (> 90%). However, the *Fy* component of the five digits in this study did not demonstrate a high PC1 value. The first PC of *Fz* comprised large contributions from the thumb, index, and middle fingers, accounting for 11 to 52%, whereas the ring and little fingers contributed less than 10% each. In contrast, the first PC of *Fy* contained a variable contribution from the thumb to the little finger. For example, the thumb accounted for 14% of PC1, but the standard deviation was more than twice its PVAF. Therefore, good synergetic performance was not observed in the *Fy* of the five digits. Future digit force studies should use PCA to analyze the strength of coordination in various force directions.

### Limitations

Although these findings enhance the understanding of cylindrical grasping kinetics, this study has several limitations. First, the cylindrical grasping simulated in the study was different from the commonly performed movement, because the participants were instructed to touch the apparatus using only their digits (that is, no palm contact). Additionally, the finger restrictions in this study were not only used to test the concept of error compensation, but also to mimic the conditions of missing fingers. However, the experimental conditions used in this work did not necessarily represent finger amputations and injuries accurately. Additional studies should focus on this issue, because synergy patterns are crucial for a clear understanding of the concept of coordination. The use of a three-axis accelerometer is strongly suggested in future works to precisely determine the phases as well as the accuracy of controlled performances during the related tasks. Moreover, this study did not consider the frictional characteristics between the contact surfaces of the simulator and the finger pads. Both perceptions of the physical characteristics of the grasped object and of changes in skin texture could affect the efficiency of force modulation. Therefore, another limitation of the present study is the lack of measurement of skin frictional properties among the participants. Finally, this study did not compare the findings obtained from the cylindrical grasp via the novel simulator and the prismatic grasp which was used in most previous investigations. Further works should also explore the differences in finger performance between these two similar but also distinct grasps.

## Conclusion

This study examined the digit force synergy in cylindrical grasp movements under both normal and anti-gravity forces. The results show that the force synergy performance can be described using normal force representation in the HSP (static condition) and the FIP (dynamic condition). In this study, specific force distribution patterns, error compensation, higher correlation coefficient values, and greater PC1 demonstrated good synergistic performance. Identifying the synergy pattern of digit forces in an anti-gravity direction was difficult due to the significant variability among participants. Therefore, it is recommended that future studies focus on normal force vectors to analyze coordination parameters. The correlation coefficients and PCA provided good representations of the coordination strength of digit forces. Additional studies should focus on force synergy in other daily tasks, because it is a crucial issue in finger coordination. For example, a greater number of hand postures should be examined to construct a more comprehensive model. Regarding the experimental design, different practice times for participants to adopt amputation-like postures could be used to determine hand coordination performance.
